# Crimean-Congo Hemorrhagic Fever in Turkey

**DOI:** 10.3201/eid1008.030928

**Published:** 2004-08

**Authors:** S. Sami Karti, Zekaver Odabasi, Volkan Korten, Mustafa Yilmaz, Mehmet Sonmez, Rahmet Caylan, Elif Akdogan, Necmi Eren, Iftihar Koksal, Ercument Ovali, Bobbie R. Erickson, Martin J. Vincent, Stuart T. Nichol, James A. Comer, Pierre E. Rollin, Thomas G. Ksiazek

**Affiliations:** * Karadeniz Technical University, School of Medicine, Trabzon, Turkey;; † Marmara University School of Medicine, Istanbul, Turkey;; ‡ Centers for Disease Control and Prevention, Atlanta, Georgia, USA

**Keywords:** hemorrhagic fever virus, Crimean-Congo hemophagocytic syndrome, infection-associated, research

## Abstract

In 2002 and 2003, a total of 19 persons in Turkey had suspected cases of Crimean-Congo hemorrhagic fever (CCHF) or a similar viral infection. Six serum samples were tested; all six were found positive for immunoglobulin M antibodies against CCHF virus. Two of the samples yielded CCHF virus isolates. Genetic analysis of the virus isolates showed them to be closely related to isolates from former Yugoslavia and southwestern Russia. These cases are the first of CCHF reported from Turkey. Eighteen patients handled livestock, and one was a nurse with probable nosocomial infection. The case-fatality rate was 20% among confirmed CCHF case-patients (1 of 5 patients), and the overall case-fatality rate was 11% (2 of 19 patients). In addition to previously reported symptoms and signs, we report hemophagocytosis in 50% of our patients, which is the first report of this clinical phenomenon associated with CCHF.

## Abstract 2

Nineteen cases of suspected Crimean-Congo hemorrhagic fever reported from Turkey.

Crimean-Congo hemorrhagic fever (CCHF) is an acute illness affecting multiple organ systems and characterized by extensive ecchymosis, visceral bleeding, and hepatic dysfunction; and it has a case-fatality of 8% to 80% (1). CCHF virus (CCHFV) (genus *Nairovirus*, family *Bunyaviridae*) is transmitted to humans by bites of infected ticks (several species of genus *Hyalomma*). CCHFV has also been transmitted to patients or viremic livestock through contact with blood or tissue (1). Epidemics of CCHFV have previously been reported from Eastern Europe, Africa, and central Asia (2–8). Many cases have been reported from the countries around Turkey, including Albania, Iran, Iraq, Russia, and the former Yugoslavia (7,9–12). Although serologic evidence indicated the existence of CCHFV in Turkey several decades ago (13), no clinical cases have been documented. We describe 19 patients from the eastern Black Sea region with hemorrhagic fever compatible with CCHF, who were admitted to Karadeniz Technical University Hospital during the spring and summer of 2002 and 2003.

## Patients and Methods

### Patients

Several patients in May through July 2002 and 2003 were referred from surrounding county hospitals to our hematology unit with varying degrees of fever and hematologic manifestations. All of the patients had similar clinical and laboratory findings, including fever, petechiae, headache, abdominal pain, nausea, vomiting, liver enzyme elevations, and cytopenia. Bone marrow aspiration and routine serologic tests excluded hematologic malignancies and known viral or bacterial infections. Serum samples from several patients admitted in 2003 were stored at –80°C for further diagnostic testing for a possible hemorrhagic fever agent.

### Laboratory Testing

Serum samples from seven patients were sent to Special Pathogens Branch, Centers for Disease Control and Prevention, Atlanta, GA (CDC) for testing. Only six samples from five patients were available in sufficient volume. After we considered possible hemorrhagic fever viruses in the region, we performed immunoglobulin (Ig) M and IgG enzyme-linked immunosorbent assay (ELISA), using inactivated native CCHFV (Strain IbAr 10200) antigens grown in Vero E6 cells on serum samples (14). A test developed to detect CCHF viral antigens was also performed (15). Virus isolation attempts from the serum samples were conducted under biosafety level 4 conditions with Vero E6 cells.

For virus genetic detection and analysis, serum samples or infected Vero E6 cells were combined with Tripure Isolation Reagent (Roche Applied Science, Indianapolis, IN) in a ratio of 1:5 and incubated at room temperature for a minimum of 10 min. Total RNA was isolated by using the RNaid Kit following manufacturer's recommendations (Qbioene Inc., Carlsbad, CA), and the extracted RNA was resuspended in 50 µL H_2_O. Five microliters of the RNA was used in a 50-µL reverse transcription (RT) reaction with the Access RT-PCR System (Promega Biosciences, San Luis Obispo, CA). The primers that enabled the amplification of nucleocapsid-coding sequence (S segment) were previously described as was the polymerase chain reaction (PCR) method used, with slight modifications (16). Briefly, separate RT was performed by using CCHF-F2 primer at 42°C for 1 h. Ten microliters of the RT reaction was subsequently used in a 50-µL PCR reaction with FastStart Taq DNA Polymerase with GC-rich solution (Roche) and primers CCHF-F2 and CCHF-R3. The temperature profile for the PCR reaction was as follows: 2 min at 95°C (36 cycles of 1 min at 95°C and 1 min at 45°C), 2 min at 72°C, and a final elongation of 10 min at 72°C. Amplified DNA was analyzed by using a 1% low-melt agarose gel, and bands corresponding to 536-bp products were purified by using the Qiagen Gel Extraction Kit (Qiagen, Valencia, CA). Sequencing of both DNA strands was performed by using primers CCHF-F2 and CCHF-R3 in a BigDye Terminator v3.1 reaction on the 3100 Genetic Analyzer (Applied Biosystems, Foster City, CA). The obtained sequences were analyzed with Sequencer (Gene Codes Corporation, Ann Arbor, MI).

## Results

Serologic test results for hepatitis A, B, and C viruses (HAV, HBV, and HCV); herpes viruses; and HIV and PCR for HBV DNA and HCV RNA were negative. Although malaria does not exist in these provinces, peripheral blood smear examinations confirmed these specimens to be negative for *Plasmodium*. Bacterial blood cultures were negative in all patients. Serologic tests for *Brucella* and *Leptospira* were also negative in all patients. Samples were negative for anti-Alkhurma virus IgM, and IgG. Specific testing for CCHFV antigen detection, IgG and RT-PCR tests were negative for the six specimens from the five patients. However, all six specimens were positive for IgM antibodies reactive with CCHFV antigen. CCHFV (CDC, Special Pathogens numbers: 200310845 and 200310849) were isolated from two of the patients.

**Figure 1:**
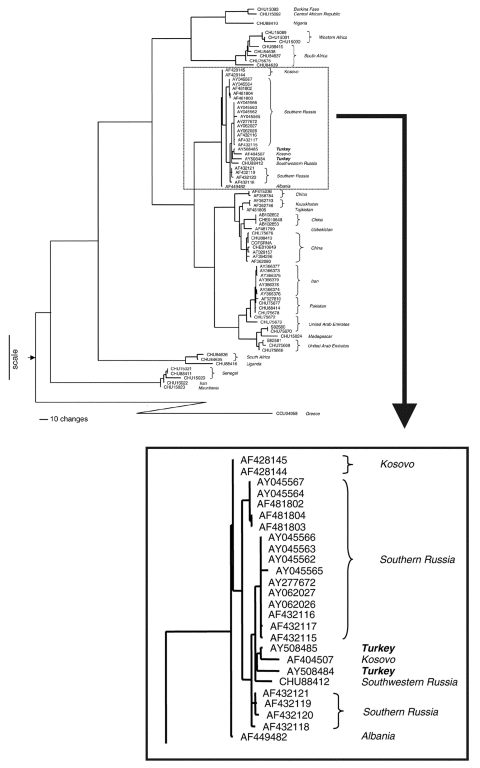
Phylogenetic analysis of Crimean-Congo hemorrhagic fever virus (CCHFV) genetic difference. Maximum parsimony analysis of the aligned sequences of a 488-nt region of CCHFV S segments and the equivalent genome region of Dugbe and Nairobi sheep disease viruses. Analysis was performed with the heuristic search method with stepwise addition, tree bisection-reconnection branch swapping, and transversions; transitions were weighted 4:1. The graphic representation of the results was outgroup rooted by using the Dugbe (GenBank accession no. AF434161, AF434162, AF434163, AF014014, AF434164, AF014015, AF434165) and Nairobi sheep disease virus (AF504293) S segment nucleotide sequences. The node attaching the outgroup to the CCHFV tree topology is shown by the arrow at the base of the tree. Horizontal distances within the CCHFV part of the tree are proportional to nucleotide steps (see scale bar), separating virus taxa and nodes. Vertical and diagonal lines are for visual clarity. Each virus sequence is indicated by the corresponding GenBank accession number. The two CCHFV sequences are in bold.

RT-PCR products of the correct predicted size (536 bp) were obtained for each of the viruses and sequenced. The resulting nucleotide sequences had high identity with previously characterized CCHFV strains, and 11 nucleotide differences were detected between the virus sequences obtained from the two patients. Comparison of the deduced amino acid sequences indicated that no amino acid differences existed between the two virus strains. Detailed genetic comparison was performed by using the CCHFV S segment sequences available from GenBank. The analysis indicated the close relatedness of the Turkish CCHFV isolates to CCHFV strains from Russia and Kosovo, with 97%–98% and 100% identity at the nucleotide and protein levels respectively (data not shown). A comprehensive phylogenetic analysis (Figure 1) by using PILEUP (Wisconsin Package Version 10.2, Genetics Computer Group, Inc.), followed by PAUP4.0b10 (Sinauer Associates Inc., Sunderland, MA, USA), showed that the Turkish CCHFV isolates clustered closely with the CCHFV strains from southwest Russia and Kosovo. Bootstrap analysis showed the clade containing the Russian, Balkan, and Turkish CCHFV to be well supported (99%), and these viruses are clearly distinct from those in other virus clades, including the clade containing the CCHFV detected in the CCHF outbreak in neighboring Iran in 2002 (GenBank accession no. AY366373–9).

**Figure 2:**
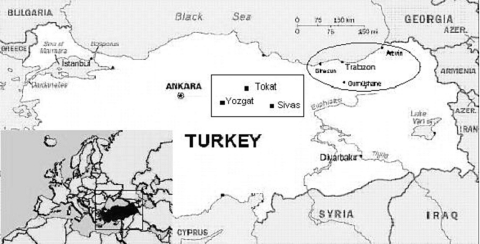
Geographic distribution of patients with Crimean-Congo hemorrhagic fever (CCHF), Turkey, 2002–2003. Residency of the patients with CCHF infection from our series is marked in the circle. Epicenter of a concurrent outbreak presented at the recent conference in Ankara is shown as a rectangle.

**Figure 3:**
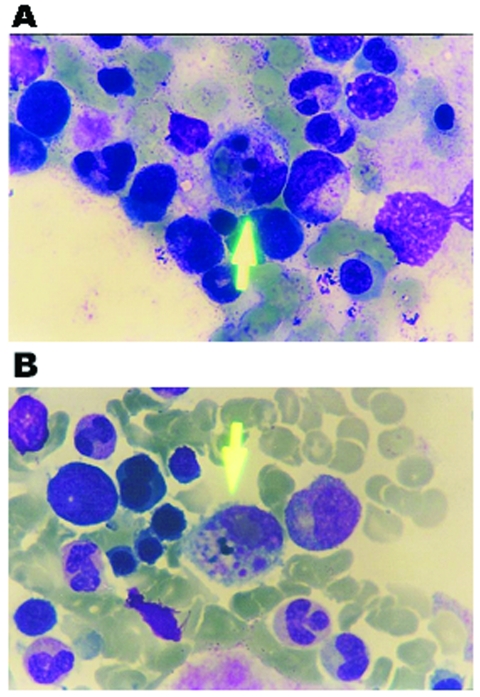
Bone marrow aspiration smear, stained with Wright, showing hemophagocytosis. A) phagocytosis of an erythrocyte and nuclear remnants by a microphange. B) phagocytosis of platelets by a macrophage.

Nineteen patients (including the five laboratory-confirmed patients) who fulfilled suspected-case criteria for CCHF of the European Network for Diagnostics of Imported Viral Diseases (ENIVD) were identified in 2002 and 2003 (17). Nine patients were admitted from May through July 2002, and 10 patients were admitted in June to July 2003. Most of the patients were female (15 female vs. 4 male), and the mean age was 42 ± 8 year. Twelve of 19 patients were from Gumushane, and the other 7 were from the neighboring cities of Giresun (4 patients), Artvin (2 patients), and Trabzon (1 patients) (Figure 2). All of them, except one, handled livestock; none of the patients described tick bites. However, six patients gave a history of removing ticks from livestock. The remaining patient was a nurse in a county hospital in Trabzon. Signs and symptoms observed in the patients are shown on the Table. The most commonly encountered signs and symptoms were malaise, fever, abdominal pain, myalgia, nausea, vomiting, petechiae, and bleeding from gingiva, nose, vagina, or gastrointestinal system. Complete blood counts showed thrombocytopenia in all patients (median 15 x 10^3^/µL, range: 1–87 x 10^3^/ µL), leukopenia in 15/19 (median 1,700/µL, range 700–5,200/µL), and anemia in 5 of 19 patients (median 13.8 g/dL, range 6.1–17.3 g/dL). Serum aspartate aminotransferase (AST) (median 693 U/L, range 178–5,220U/L), alanine aminotransferase (ALT) (median 248 U/L, range 66–1,438 U/L), and lactate dehydrogenase (LDH) (median 1,601 U/L, range 650–20,804 U/L) levels were elevated in all patients. Coagulation tests showed prolonged prothrombin time (PT) (median 13.4 s, range 12.1–18.5 s) and activated partial thromboplastin time (aPTT) (median 34.9 s, range 30.2–59.1 s) in 7 of 19 patients. Fibrinogen was decreased and D-dimer was elevated in one patient with suspected CCHF, which indicated disseminated intravascular coagulation. Fibrinogen and D-dimer levels were normal in other patients. Creatine phosphokinase (CPK) levels were elevated in 14 of 19 patients (median 568 U/L, range 81–2,500 U/L). Blood urea nitrogen and creatinine (median 0.8 mg/dL, range 0.5–6.2 mg/dL) were found to be elevated in 2 of 19 patients. Hematologic malignancies were excluded after bone marrow aspiration smear and trephine biopsy in 14 patients. In 7 of 14 patients (including 2 of 5 confirmed patients), hemophagocytosis with proliferation of histiocytes in bone marrow smears was present (Figure 3).

**Table:** Signs and symptoms among clinically suspected and confirmed CCHF patients

Signs and symptoms	Confirmed cases n = 5	Suspected cases n = 14	Total (%) n = 19
Malaise	5	14	19 (100)
Fever	4	12	16 (84)
Nausea and vomiting	3	13	16 (84)
Abdominal pain	3	13	16 (84)
Petechiae-ecchymosis	5	6	11 (58)
Myalgia	4	4	8 (42)
Bleeding from various sites	1	7	8 (42)
Diarrhea	3	4	7 (37)
Lymphadenopathy	1	3	4 (21)
Hepatomegaly	1	3	4 (21)

CCHF, Crimean-Congo hemorrhagic fever.

All patients received intensive clinical supportive measures, including platelets, fresh frozen plasma, and packed erythrocyte infusions, when indicated. Despite supportive treatment, one confirmed and one suspected CCHF patient died. The suspected CCHF patient was a nurse who had a history of taking care of similar clinical patients in a county hospital in Trabzon. She died of intraabdominal and pulmonary hemorrhage. The other patient died of massive gastrointestinal bleeding. The remaining 17 patients recovered within 5 to 10 days with clinical supportive measures.

## Discussion

CCHF was first described in Crimea in 1944. In 1969, the pathogen that caused the disease was recognized to be the one responsible for febrile illnesses identified in the Congo. Since then, many human cases have been reported from different regions, namely Zaire, Uganda, Saudi Arabia, United Arab Emirates, Pakistan, European Russia, Iran, and South Africa (2–9). Additionally, sporadic cases, as well as large outbreaks, were reported from various regions, such as Kosovo and Kenya (10,12,18). Neither sporadic cases nor outbreaks have been previously reported from Turkey. All of the five patients' serum samples tested were found to be positive for IgM antibodies for CCHFV. Findings from the RT-PCR, antigen detection, and IgG tests were negative. These findings are in accordance with recent infection with CCHFV in these five patients. The negative RT-PCR findings are in accordance with the presence of IgM in all the samples; we usually find that we cannot detect infectious virus or virus RNA once detectable antibody has developed. Nevertheless, on this occasion, we were able to isolate CCHFV from two of the patients. IgM and IgG antibodies are usually not detectable in early phase of illness, and they usually begin to rise during day 7–10 of infection. During the early phase, antigen detection and RT-PCR are usually the tests of choice for a sensitive laboratory diagnosis (19). All the patients were referred to our clinic, and blood samples were drawn >1 week after onset of illness.

These CCHF cases are among the first documented in Turkey. Similar cases have been reported in other provinces of eastern Turkey. Tokat, Yozgat, and Sivas seem to be the epicenter of the outbreak (Turkish Society of Clinical Microbiology and Infectious Diseases, unpub. data) (Figure 2). The cases in those areas are the subject of ongoing epidemiologic studies. No deaths were observed among the suspected CCHF patients during 2002; 2 of the 10 patients in the 2003 outbreak died of extensive visceral hemorrhages. One of the patients was a nurse in the emergency clinic of a local hospital with a possible exposure to a suspected CCHF patient. Nosocomial transmission of CCHFV through infected blood or body secretions from patients has been reported many times in the literature (12,20–22). The exact procedures performed by the nurse are not clear. She likely had an exposure to blood or infected body fluids of viremic patients affected by an unknown disease in the region. All the other patients handled livestock. In the eastern Black Sea region, women carry out most of the livestock handling, which may explain why most of the patients were female. Handling CCHF-infected animal materials, such as milk and meat, is a recognized means of infection (19) and the probable means of infection for most of our patients, since none had a reported history of tick bite. Some of our patients also gave a history of removing ticks from livestock, and this behavior has been incriminated in CCHF infections.

The most common clinical signs and symptoms reported in CCHF are fever, myalgia, dizziness, malaise, backache, headache, photophobia, nausea, vomiting, diarrhea, abdominal pain, petechiae, ecchymosis, and visceral bleeding. Most of these signs and symptoms were also observed in our patients. We observed elevated CK levels in 14 (75%) of 19 patients, including all of the confirmed CCHF patients. Elevated CK values can be explained with myositis, but the pathologic findings do not demonstrate myositis in the literature, and we did not have muscle biopsies from our patients. Rhabdomyolysis could be another explanation for elevated CK values, but urine samples were also not tested for myoglobinuria. Among those patients with high CK levels, two had acute renal failure. Elevated CK values have also been reported in some other clinical series (23).

Hemophagocytosis, which has not been reported previously in CCHFV infections, was also found in our patients. This condition can develop secondary to many viral, bacterial, fungal, parasitic, and collagen vascular diseases (24). We detected reactive hemophagocytosis in 7 (50%) of 14 patients, which suggested that hemophagocytosis can play a role in the cytopenia observed during CCHF infection. Varying degrees of cytopenia are consistently found in CCHF infection (23), but to our knowledge, this is the first study demonstrating hemophagocytosis in CCHF patients. Only two case reports demonstrate hemophagocytosis with Hantaan and Puumala viruses (genus *Hantavirus*) among all the hemorrhagic fever viruses (25,26). Excessive activation of monocytes attributable to stimulation by high levels of Th1 cytokines, such as interferon-γ, tumor necrosis factor-α, interleukin (IL)-1 or IL-6, are proposed as possible immunopathologic mechanism of hemophagocytic lymphohistiocytosis (24). Cytokine studies are lacking in CCHFV infection and are needed for a better understanding of pathogenesis of the disease caused by CCHFV.

Prolongation of PT and PTT was thought to be caused by liver damage. However, in one of our patients, disseminated intravascular coagulation was clearly demonstrated. That patient was the nurse who died with pulmonary and intraabdominal bleeding. Contributing disseminated intravascular coagulation may be associated with a poor prognosis in CCHF infection. Although disseminated intravascular coagulation has been reported previously in some CCHF cases, the exact mechanism for hemorrhage remains unknown (23,27). Of the viral hemorrhagic fevers, CCHF infection has the most florid hemorrhage and highest frequency of large ecchymoses. Besides elevated PT, aPTT, and thrombocytopenia, damage to vascular endothelium directly by the virus can lead to bleeding tendencies (27,28).

Overall laboratory findings in our patients were consistent with the findings in other CCHF case series. Liver transaminase levels were high in our patients, and AST values were generally higher than ALT values, probably attributable to concomitant muscle damage. Beside the hepatic vascular involvement and resulting infarctions in liver parenchyma, direct hepatocellular involvement may also be responsible for elevated serum aminotransferases (23,27).

Any of the following clinical pathologic values during the first 5 days of illness were found to be >90% predictive of fatal outcome in a series of South African CCHF patients: leukocyte counts <10 x 10^9^/L, platelet counts <20 x 10^9^/L, AST >200 U/L, ALT >150 U/L, aPTT >60 s, and fibrinogen <110 mg/L (23). Although most of our patients have at least one or more of the risk factors described above, the overall death rate was low at 11%. Although very high death rates are reported in some series, low death rates in our patients can be explained with better supportive care of the patients. Regional strain differences in CCHFV may also play a role in the differential death rates.

Phylogenetic analysis of virus sequence differences indicates that at least two different genetic lineages of CCHFV are circulating within this current Turkish outbreak. These closely resemble virus lineages found in Kosovo and southwestern Russia and are clearly distinct from those associated with the recent CCHF outbreak in Iran in 2002 (9). The data are most consistent with CCHF's being enzootic in the affected areas in Turkey, rather than having been introduced from Iran by infected tick or livestock movement. The virus might also have come from Russia by birds migrating with their ticks across the Black Sea. Turkey is known to be on the flight path of some birds migrating from Russia to Africa during the winter. However, a number of recognized tick vectors and reservoirs have been known to occur in the region for many years (29), and serologic data from several decades in the past support the previous existence of the virus as well (13).

Our patients are among the first with documented cases of CCHFV infection in Turkey. Recognition of dozens of cases in many provinces of Eastern Turkey during the last 2 years led to the awareness of a previously unrecognized illness in the region. In addition, we documented, for the first time, the occurrence of reactive hemophagocytic syndrome in CCHFV infection, which may be responsible for some of the clinical manifestations. Tick bite, occupational exposure to the virus from infected animals, and nosocomial exposure to patients appear to have been the major transmission routes in this outbreak.
